# Genetic Background of the Hetian Rose Inferred from SNPs in Single-Copy Nuclear Loci and Plastid Genomes

**DOI:** 10.3390/genes17101234

**Published:** 2026-10-06

**Authors:** Hui Wang, Mingwei Zhou, Yuee Xiao

**Affiliations:** Shanghai Engineering Research Center of Sustainable Plant Innovation, Shanghai Botanical Garden, Shanghai 200231, China; wanghui@shbg.org (H.W.); 13402115117@163.com (M.Z.)

**Keywords:** *Rosa* ‘Hetian’, oil-bearing roses, *Rosa gallica*, genome resequencing, population structure, plastid genome, phylogenetic analysis

## Abstract

Background/Objectives: The Hetian Rose (*Rosa* ‘Hetian’) is a locally important oil-bearing rose cultivar grown in Hetian, Xinjiang, China, but its genetic origin remains unclear. Methods: To clarify its genetic background, we sampled *R*. ‘Hetian’ together with 35 related *Rosa* species and cultivars and performed phylogenetic and genetic-structure analyses using 7478 SNPs in 1851 single-copy nuclear loci. We also reconstructed a plastid genome phylogeny to assess its maternal-lineage affinity. Results: In the nuclear SNP phylogeny, *R*. ‘Hetian’ was associated with European rose lineages rather than Chinese Hybrid Rugosa roses. Genetic-structure analyses indicated that *R*. ‘Hetian’ showed its strongest nuclear genomic affinity to Gallica roses, with weaker affinities to several other Eurasian lineages. In the plastid phylogeny, *R*. ‘Hetian’ clustered with Gallica and Alba roses, sect. *Caninae*, and European sect. *Synstylae* accessions. Conclusions: Together, these results indicate that the genetic background of *R*. ‘Hetian’ is closely related to European Old Garden Roses. A Gallica-related lineage may have made a major contribution to its genomic background. Historical genetic exchange with other Eurasian *Rosa* lineages may also have contributed to its present genomic composition.

## 1. Introduction

China is one of the major rose-producing regions, and the Hetian Rose (*Rosa* ‘Hetian’ is an important commercial oil-bearing rose cultivar in China. The Hetian Rose, also known as “Xiaozhi Meigui”, is mainly cultivated in Hetian (Hotan) Prefecture, Xinjiang Uygur Autonomous Region. It is cultivated over approximately 5700 ha and yields about 15,000 tons of fresh flowers annually [1]. Moreover, the Hetian Rose is widely used as drought-tolerant vegetation for desertification control on the south edge of the Taklamakan Desert. Despite its economic and ecological importance, the origin and genetic background of this cultivar remain poorly understood, mainly because detailed historical records are lacking. This constrains standardized production, germplasm conservation, and further utilization of this cultivar.

Over the past two decades, parentage testing, phylogenetic reconstruction and population genetic analysis based on molecular markers have been used to investigate the evolution and domestication history of several rose groups, such as Damask roses [2], Noisette roses [3], Bourbon roses [4], China roses [5], an ancient Chinese hybrid Rugosa called *Rosa* ‘Kushui’ [6], and many edible roses in China [7]. However, hybridization and polyploidization are extremely prevalent in the genus *Rosa*. Therefore, analyses based on plastid genes or a few nuclear loci may not adequately reflect the complex evolutionary and domestication histories of roses [8].

Whole-genome sequencing (WGS) and genome-scale analyses now provide greater power to resolve these complex relationships. The hybrid origins of ten rose species, including economically important *R. gallica* and *R.* × *damascena*, were investigated using 92 single-copy nuclear loci and 4 plastid genes [8]. Phylogenetic analysis based on haplotype-resolved genomic data resolved the evolutionary origin of pentaploid *Canine* rose, which was a mystery for more than 100 years [9]. Likewise, the allotetraploid *R. gallica* was inferred to have originated from ancient hybridization between *Cinnamomeae* lineage and European *Synstylae* lineage using similar methods [10]. Moreover, the haplotype-resolved chromosome-scale genome assembly of *R*. ‘Samantha’, a tetraploid Hybrid Tea, revealed the genomic composition of a modern rose [11].

The cultivation history of the Hetian Rose can be traced back approximately 300 years, although detailed historical records are lacking, and this cultivar has not been documented outside China. A double-flowered rose depicted in a mural from the ancient Milan site, dating to approximately 1600 years ago, resembles the Hetian Rose. Because of its economic importance and unique distribution, several studies have partially investigated the genomic background of *R.* ‘Hetian’ [1,12,13,14]. Phylogenetic analyses based on several nuclear and plastid loci suggested that *R.* ‘Hetian’ is related to *R.* × *damascena*, and subsequently underwent geographical isolation, thereby forming an independent, endemic cultivar. More evidence is needed to test this interpretation.

In this study, we broadly sampled *Rosa* species and cultivars potentially related to *R*. ‘Hetian’. Whole-genome resequencing data were used to analyze SNP variation in single-copy nuclear loci for phylogenetic and genetic-structure analyses. In addition, we reconstructed a plastid genome phylogeny to assess the maternal-lineage affinity of *R*. ‘Hetian’. By integrating nuclear and plastid genomic evidence, this study aims to clarify the genetic background of *R*. ‘Hetian’ and provide a genomic basis for its identification, conservation, and utilization.

## 2. Materials and Methods

### 2.1. Sample Collection

*Rosa* ‘Hetian’ and 35 related *Rosa* species and cultivars were included in this study, comprising five Gallica roses (*R. gallica* and its cultivars), eight Damask roses (*R.* × *damascena*), two Alba roses (*R*. × *alba*), two Centifolia roses (*R*. × *centifolia*), six Hybrid Rugosa roses (*R. rugosa* and its cultivars or hybrids), and twelve wild *Rosa* species (six from sect. *Cinnamomeae*, four from sect. *Synstylae*, and two from sect. *Caninae*). Most accessions are cultivated and maintained in the research nursery of Shanghai Botanical Garden. Young leaves were collected and dried in silica gel prior to DNA extraction. Detailed sample information is provided in Table 1.

### 2.2. DNA Extraction and Sequencing

Genomic DNA was extracted from dried leaves using the cetyltrimethylammonium bromide (CTAB) method [15]. Sequencing libraries with an average insert size of approximately 350 bp were prepared using the Nextera XT DNA Library Preparation Kit (Illumina, San Diego, CA, USA). After quality control, paired-end 150 bp sequencing was performed on an Illumina NovaSeq 6000 platform by Shenzhen Huitong Biotechnology Co., Ltd. (Shenzhen, China). Sequencing was targeted at approximately 30× coverage relative to the estimated haploid genome size (~0.5 Gb).

### 2.3. Plastid Genome Assembly and Phylogenetic Analysis

Raw reads were quality-filtered using fastp v0.19.7 [16]. Clean reads were de novo assembled using SPAdes v3.14.1 with k-mer sizes of 21, 45, 65, 85, and 105 [17]. Bandage v0.8.1 was used to inspect the assembly graph, remove residual contigs, and circularize the plastid genome [18]. To assess assembly quality, clean reads were mapped back to the assembled plastid sequence using BWA-MEM, and sequencing depth and coverage were calculated using SAMtools depth [19,20]. The resulting plastid genome sequences were annotated using PGA, with GenBank accession OR381389 as the reference [21].

Plastid genome sequences were aligned using MAFFT v7.525, and poorly aligned regions were removed using Gblocks v0.91b [22,23]. A maximum-likelihood phylogeny was reconstructed using IQ-TREE v3.0.1 under the TVM + F + R5 model with 1000 bootstrap replicates [24]. *Rosa hugonis* and *Fragaria* × *ananassa* were used as outgroups.

### 2.4. SNP Calling and Filtering

Given the complex history of polyploidization and hybridization in *Rosa*, we focused on SNPs located within previously identified single-copy orthologous (SCO) nuclear loci rather than genome-wide SNPs to reduce potential biases associated with paralogous mapping. A set of 1856 SCOs from *Rosa chinensis* ’Old Blush’ was used as a reference [8]. The loci ranged from 300 to 550 bp in length, with a total length of 663,805 bp. BWA-MEM v.2.2.1 [19] was used to build the index from the reference sequence and align the cleaned reads from each sample to the reference sequences. GATK was used for SNP calling and preliminary filtering (QD < 2, MQ < 40, FS > 60, SOR > 3, QUAL < 30, MQRankSum < −12.5, ReadPosRankSum < −8) [25]. The variants were filtered using VCFtools (minQ 30, min-meanDP 5, max-missing 0.7, maf 0.05; only SNPs with two alleles were preserved), then linked sites were removed using PLINK (indep-pairwise 50 10 0.2) to avoid linkage disequilibrium [26,27]. Ploidy of each species or variety followed Cheng et al. (2025), Zhang et al. (2024) and helpmefind.com [11,28].

### 2.5. Phylogenetic and Genetic-Structure Analyses

A maximum-likelihood tree including 36 *Rosa* accessions and the outgroup was inferred from the SNP dataset using FastTree v2.1.11 under the generalized time-reversible (GTR) model [29]. The nuclear SNP tree was rooted according to relationships inferred from the plastid phylogeny and previous phylogenomic studies [8,28].

Principal component analysis (PCA) was performed using GCTA v1.94.0 with default settings [30]. Genetic structure was estimated using ADMIXTURE based on the binary PLINK dataset [31]. Analyses were performed for K = 2–12. The K value with the lowest cross-validation (CV) error was identified, and higher K values were additionally examined to evaluate finer-scale genetic structure in relation to the taxonomic groups represented in the sampling.

## 3. Results

### 3.1. Read Assembly and SNP Calling

The mean sequencing depth among samples ranged from 11.441× to 40.625×, depending on ploidy, and approximately 78–100% of the reference sequence was covered (Table S1). A total of 19,638 SNP sites were identified across the 36 *Rosa* accessions, of which 92.93% were biallelic. After linkage-disequilibrium pruning, 7478 SNPs distributed across 1851 loci were retained for downstream analyses. The mean sequencing depth at retained SNPs was 20.01×, and the overall missing-genotype rate was 8.04%. Missing-genotype rates ranged from 3.41% to 24.31% among accessions and from 0% to 27.78% among SNP sites.

The mean sequencing depth of the plastid genomes exceeded 100×. Plastid genome lengths ranged from 156,603 to 157,359 bp (Table S1). A total of 135 annotated gene copies, including 90 protein-coding gene copies, were identified in each plastid genome (Figure S1).

### 3.2. Phylogenetic Analysis

**Figure 1:**
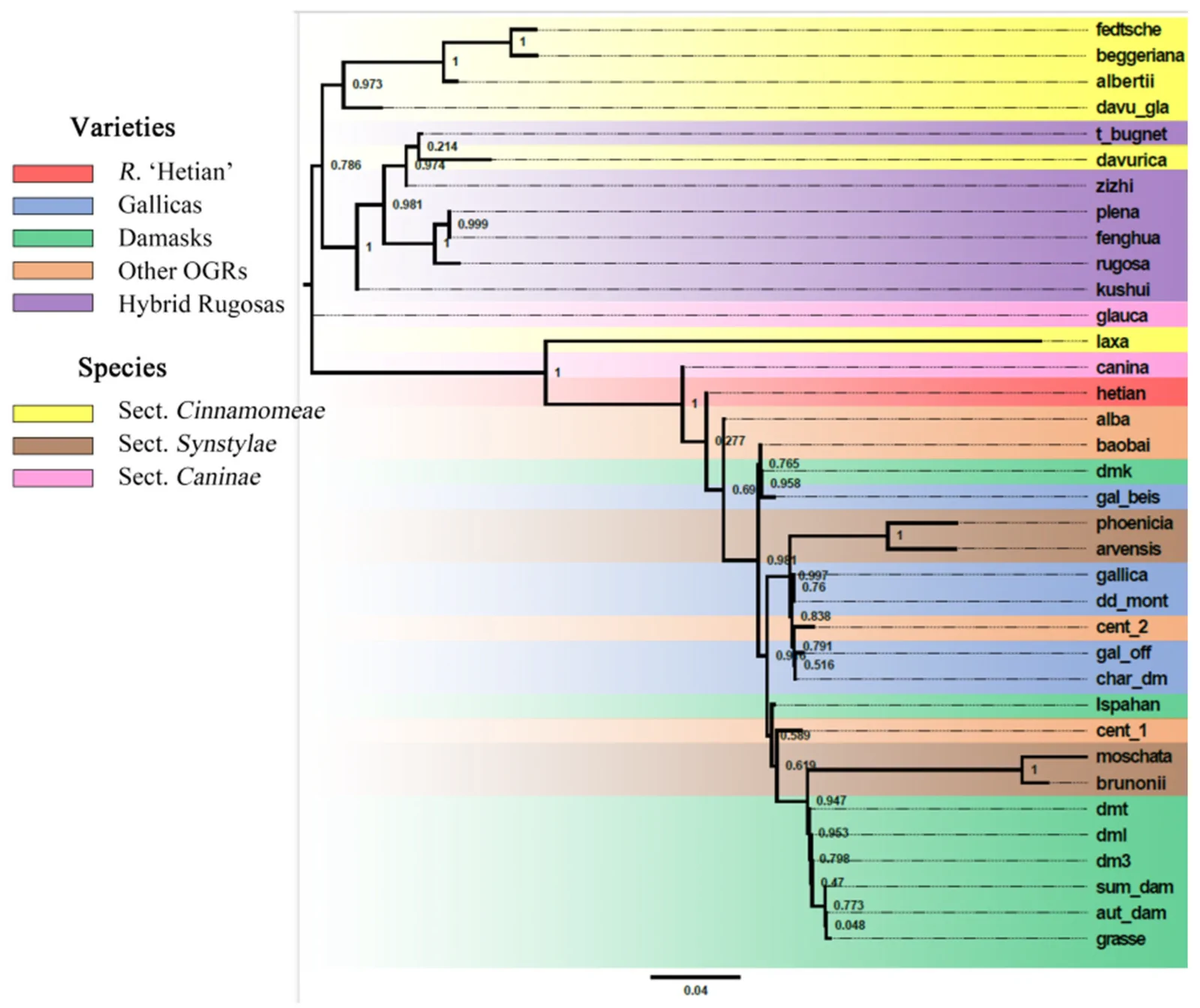
Maximum-likelihood phylogenetic tree of *Rosa* ‘Hetian’ and related roses based on genomic SNPs from 7478 single-copied nuclear loci.

The maximum-likelihood phylogenetic tree based on 7478 SNPs is shown in Figure 1. Species from section *Cinnamomeae*, except for *R. laxa*, formed a monophyletic clade comprising two subclades: one containing *R. rugosa*, *R. davurica* and their varieties or hybrids (namely Hybrid Rugosa roses), and the other containing species distributed in northwestern China and Central Asia. All Gallica roses except for *R.* ‘Bella Isis’ formed a monophyletic group, with European *Synstylae* species (*R. arvensis* and *R. phoenicia*) as its sister group. Likewise, most Damask roses formed a monophyletic clade, whereas *R*. ‘Ispahan’ and dmk were excluded. The *R. brunonii*–*R. moschata* clade was the sister group of the principal Damask clade. *Rosa* ‘Hetian’, as well as *R.* ‘Bella Isis’ (Gallica), *R.* ‘Ispahan’, dmk (Damask), cent_1 (Centifolia), Alba roses (*R.* × *alba* and Baobai) and section *Canine* species (*R. canina* and *R. glauca*) fell outside the major Gallica and Damask clades and did not form a monophyletic group.

**Figure 2:**
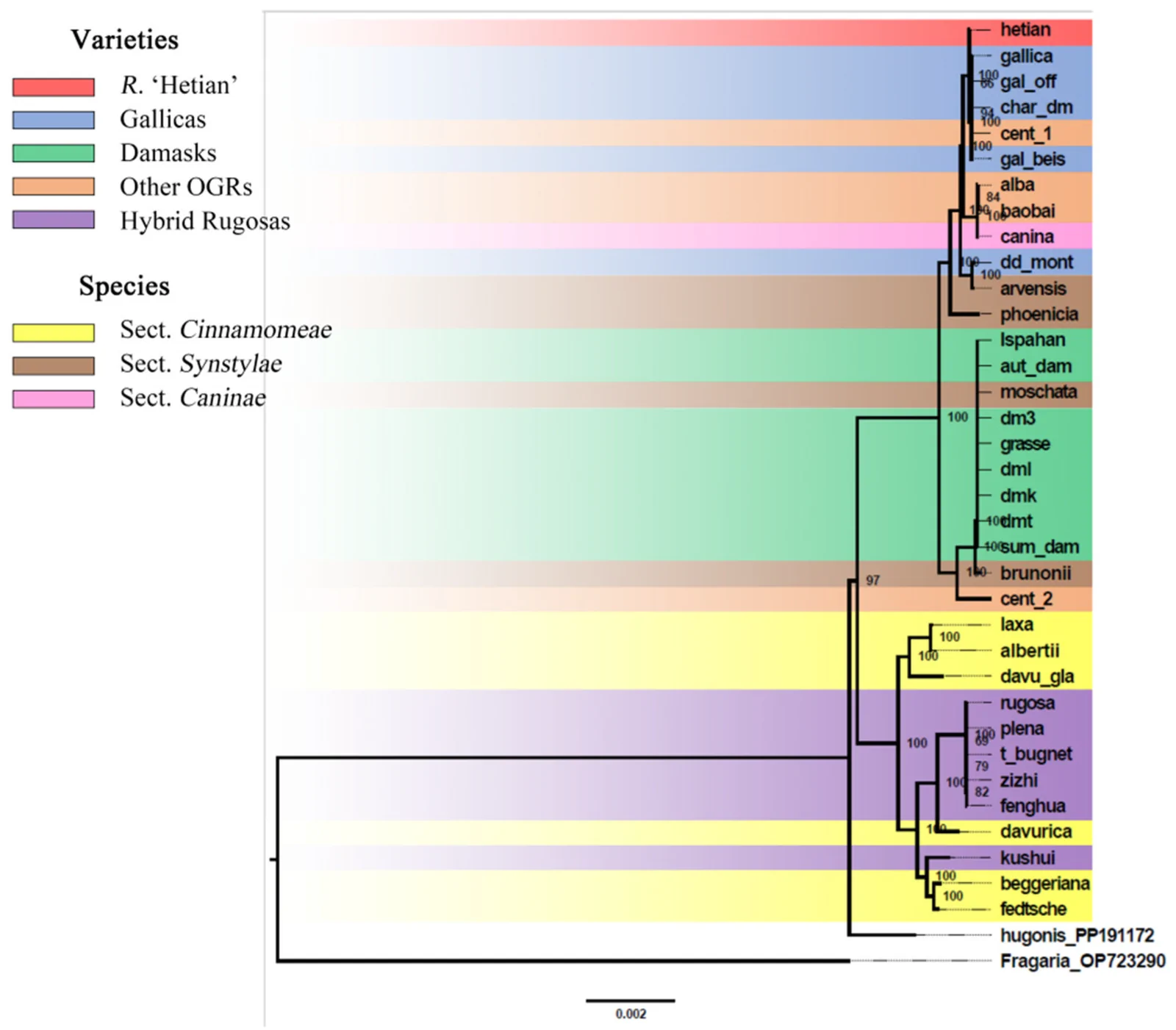
Maximum-likelihood phylogenetic tree of *Rosa* ’Hetian’ and related roses based on plastid genomes.

In the plastid genome phylogeny (Figure 2), *Rosa* ‘Hetian’, cent_1, the sampled Gallica and Alba roses, sect. Caninae species, and European sect. Synstylae species formed a clade. The sister group comprised all sampled Damask roses, cent_2, *R. brunonii*, and *R. moschata*. Species and cultivars of sect. *Cinnamomeae* formed a separate clade.

### 3.3. Genetic Structure

**Figure 3:**
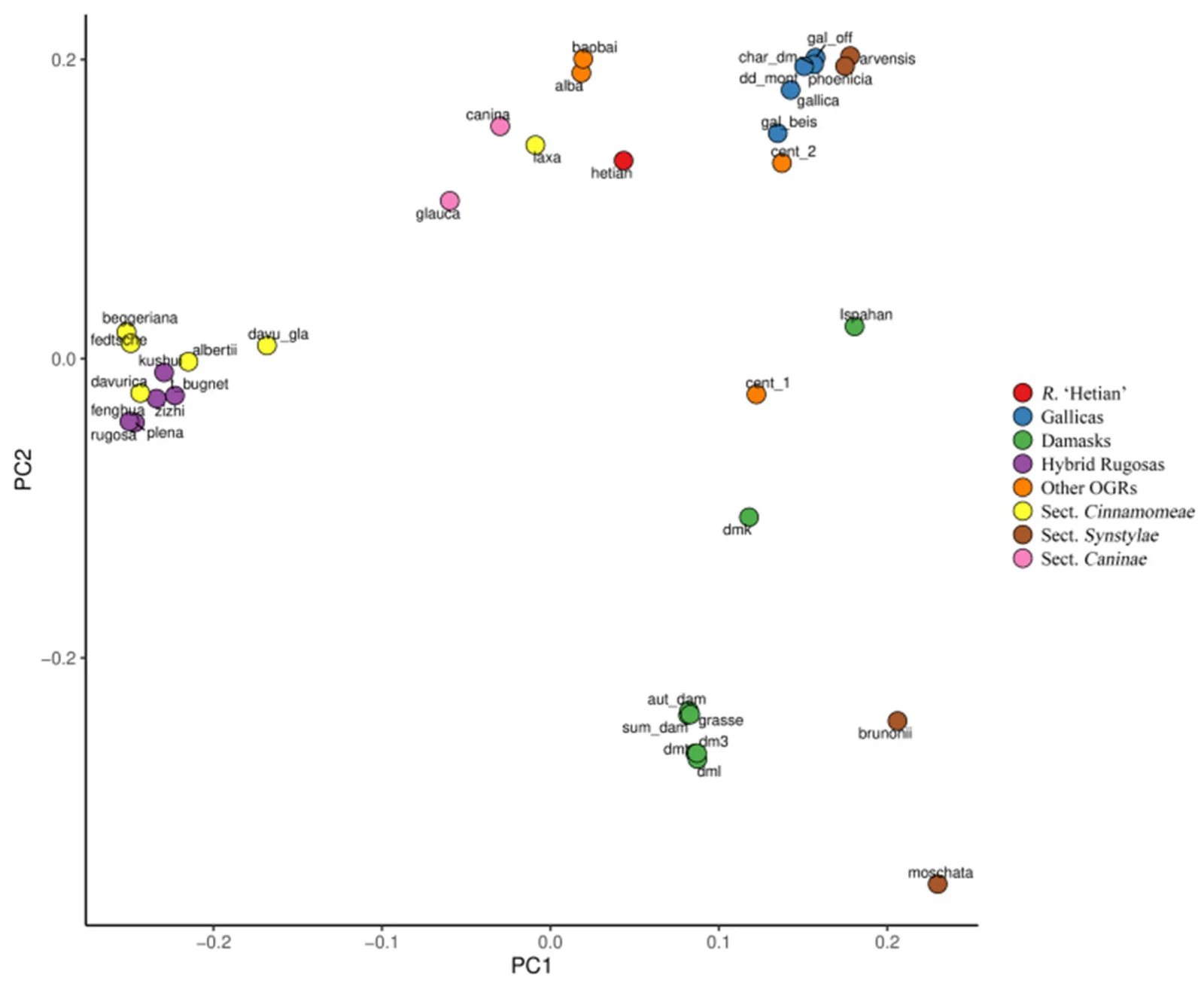
The PCA plot of 36 *Rosa* accessions, including *R.* ‘Hetian’. The classification of each sample is represented by color. OGRs = Old Garden Roses.

The PCA results are shown in Figure 3. The sampled Gallica roses, Damask roses (except *R*. ’Ispahan’ and dmk), and *Cinnamomeae* species formed three distinct clusters. *R. arvensis*–*R. phoenicia* and *R. brunonii*–*R. moschata* were positioned near the Gallica and Damask groups, respectively. *R*.’Hetian’ occupied an intermediate position among these clusters but was positioned closest to the Gallica accessions. Cent1, dmk, and R. ‘Ispahan’ were positioned between the Gallica and Damask groups, whereas the Alba roses, sect. *Caninae* species, and *R. laxa* were positioned between the Gallica and *Cinnamomeae* groups.

**Figure 4:**
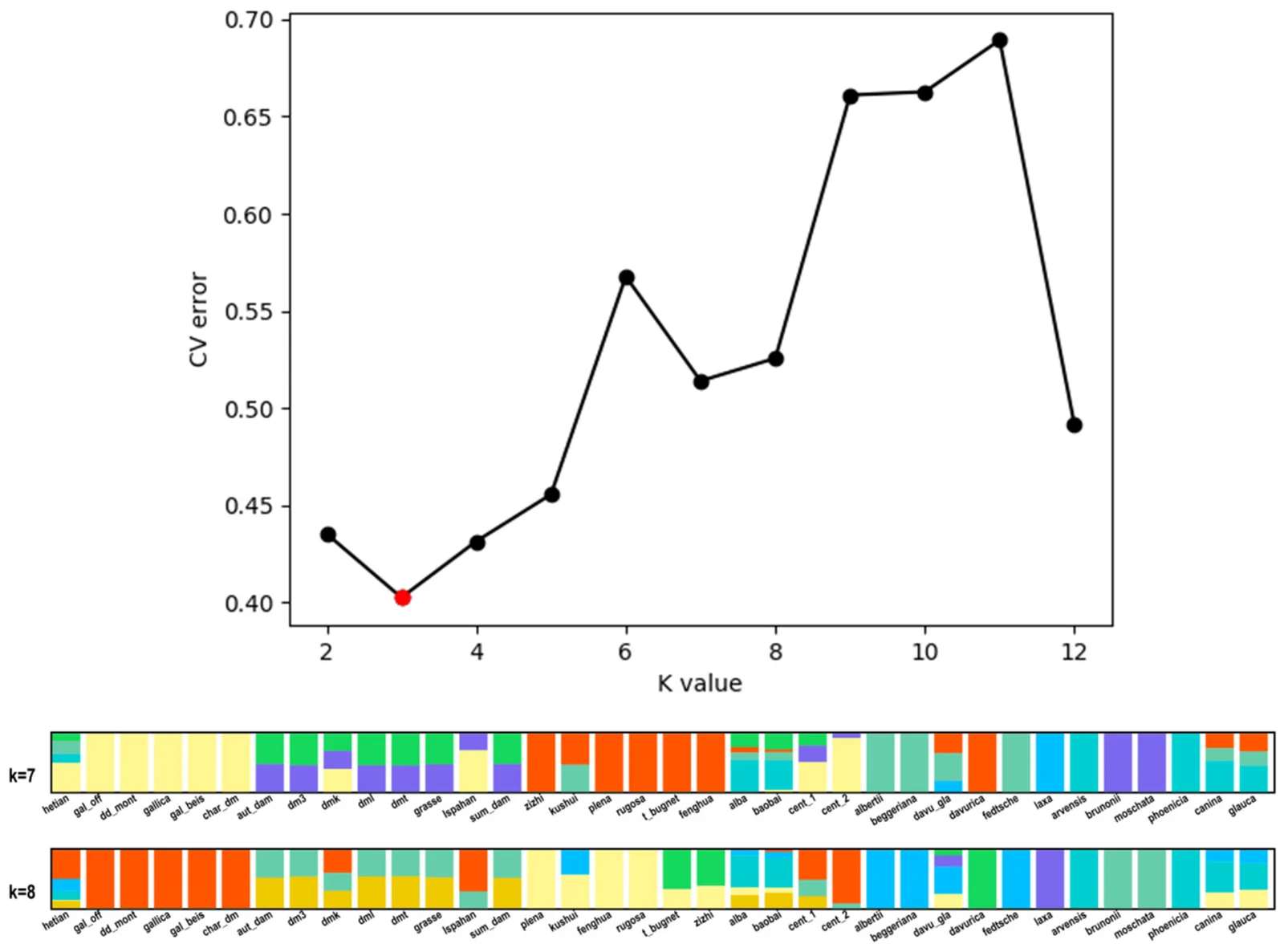
Population structure of *Rosa* ‘Hetian’ and related rose species or varieties: Above: the CV-error curve. The CV-error is lowest at k value = 3 (red dot). Below: The ADMIXTURE bar plot with k value = 7, 8.

In the ADMIXTURE analysis, the lowest CV error occurred at K = 3, with additional decreases at K = 7, 8, and 12 (Figure 4). We additionally examined K = 8 because it provided finer differentiation among several major taxonomic groups represented in our sampling; K = 8 should therefore be regarded as a biologically informative higher-resolution representation rather than the statistically optimal model based solely on CV error. The bar plot is shown in Figure 4 and Figure S2. At K = 8, most wild species were dominated by a single ancestry component. Exceptions included *R. canina*, *R. glauca*, and *R. davurica* var. *glabra*. The genetic components of each type were named Gal (*R. gallica*), Rug *(R. rugosa*), Dar (*R. davurica*), CinW (*R. beggeriana*, *R. albertii* and *R. fedtschenkoana*), SynA (*R. arvensis*, *R. phoenicia*) and SynB (*R. brunonii*, *R. moschata*) in this article. Six of the eight Damask rose varieties shared very similar genetic components, comprising the unique component of Damasks (Dam) and SynB. For the two exceptions, Dmk contained additional Gal, while *R.* ‘Ispahan’ contained only Gal and SynB components. The genetic component of *R*. ‘Hetian’ was mainly Gal, but Dam, CinW and SynA were also included. For Hybrid Rugosa roses, the genetic component of *R. rugosa* ‘Plena’ and *R. rugosa* ‘Fenghua’ contained the only Rug component, while *R*. ‘Zizhi’ and *R*. ‘Thérèse Bugnet’ contained both Rug and Dar, whereas *R*. ‘Kushui’ contained Rug and CinW components.

## 4. Discussion

Oil-bearing roses are fragrant *Rosa* species and cultivars grown primarily for essential-oil production. Such roses have been cultivated in both China and other regions (e.g., Europe and Western Asia) for centuries, but different species were selected in each region. In China, most oil-bearing roses are derived from *Rosa rugosa*, a species native to east Asia. In other regions, European Old Garden Roses were cultivated for oil production. European Old Garden Roses include four main types: Gallicas (varieties of *R. gallica*), Damasks (*R.* × *damascena*), Centifolias (*R.* × *centifolia*) and Albas (*R.* × *alba*). Unlike modern roses, which have largely been developed through deliberate hybridization, these old rose varieties largely arose through spontaneous mutation and natural hybridization. Moreover, historical records concerning the discovery, cultivation, and dispersal of old roses are usually scarce. Therefore, the origin of many old roses remains unsolved. Here, we applied whole-genome resequencing combined with phylogenetic and population genetic analysis to investigate the origin of *Rosa* ’Hetian’.

### 4.1. Genomic Affinity of Rosa ‘Hetian’ with European Old Garden Roses

To clarify the genetic background of the Hetian Rose, we broadly sampled rose species and cultivars that might be genetically related to *R*. ‘Hetian’. The sampling included (1) Chinese Rugosa roses, which were traditional oil-bearing roses cultivated in other regions of China; (2) representative varieties of all four types of European Old Garden Roses; and (3) wild *Rosa* species previously suggested as ancestors of oil-bearing roses, or occurring in Xinjiang.

Complex ploidy level is a main difficulty in phylogenetic and population genetic analysis of genus *Rosa*. Our samples included species and cultivars with different ploidy levels (Table S1). Therefore, we use a set of single-copy orthologous (SCO) nuclear loci as the reference sequence, which would avoid misidentifying paralogues as orthologues in polyploid species. Additionally, only biallelic SNPs were retained to simplify downstream analysis, so multiallelic loci and allelic frequency were not considered. However, over 90% of the SNPs were biallelic in our results, indicating that loss of informative sites was reduced.

In the phylogenetic tree based on SNP data, the other three major Chinese oil-bearing roses, *R. rugosa* ‘Fenghua’, *R. rugosa* ‘Plena’ and *R.* ‘Kushui, clustered with their ancestors, *R. rugosa* and other members of section *Cinnamomeae*. However, *R.* Hetian was not included in this group. Instead, it was placed within the clade comprising European Old Garden Roses and related species, such as Gallicas, Damasks, Albas, *R. canina*, *R. moschata* and *R. phoenicia*. Similarly, in the plastid genome tree, *R.* ‘Hetian’ clustered with Gallicas, Albas, *Caninae* and European *Synstylae* species. Previous studies revealed that allotetraploid *R.gallica* and allopentaploid *Caninae* species originated from ancient hybridization between European *Synstylae* species and other lineages, consistent with our results [2,8,9,10]. Therefore, *R.* ‘Hetian’ was not derived from *R. rugosa* like other Chinese oil-bearing roses. Otherwise, it shows greater genetic affinity to European old roses like Gallica roses and Damask roses. These results are consistent with previous phylogenetic analyses based on several nuclear and plastid loci [1,12,13,14].

Notably, the phylogenetic position of *R*. ‘Hetian’ differed between the nuclear SNP and plastid genome trees. Because plastid genomes in *Rosa* are generally maternally inherited, whereas nuclear genomes are inherited biparentally, such cytonuclear discordance may reflect historical hybridization or genetic exchange between divergent rose lineages. However, incomplete lineage sorting and differences in phylogenetic resolution cannot be excluded.

### 4.2. Genomic Affinity of Rosa ‘Hetian’ to Gallica Roses

In the PCA plot, the three major groups of oil-bearing roses—Gallicas, Damasks, and Hybrid Rugosa roses—formed distinct clusters. The Gallica and Damask groups were relatively close along PC1 but separated along PC2, whereas Hybrid Rugosa roses were distinct along both dimensions and grouped with other *Cinnamomeae* species. *Rosa* ‘Hetian’ occupied an intermediate position but was closest to the Gallica accessions. This pattern supports a stronger genome-wide affinity of *R*. ‘Hetian’ to the sampled Gallica roses, with weaker affinities to other lineages; however, PCA proximity alone does not demonstrate direct ancestry.

In the ADMIXTURE result with k value set as 8, Hybrid Rugosas, Gallicas and Damasks formed three distinct groups, although several varieties contained components from other groups. The ancestry profile of *R*. ‘Hetian’ was dominated by the Gal component, with smaller contributions from components associated with Damask roses, some *Cinnamomeae* species (native to Central Asia—Xinjiang) and European *Synstylae* species. No component related to *R. rugosa* was detected. Therefore, we infer that a Gallica-related lineage may have contributed substantially to the ancestry of *R*. ‘Hetian’, but other species and varieties from Europe to western China may also have contributed to its ancestry. Additionally, The ADMIXTURE patterns observed for Damask roses, *R. canina*, *R*. ‘Kushui’, *R*. ‘Zizhi’ and *R*. ‘Thérèse Bugnet’ are consistent with previous studies based on genome data or molecular markers, as well as recorded pedigrees [2,6,7,8,9,10,32]. These consistencies provide additional support for our interpretation.

Most cultivated rose varieties are clonally propagated; therefore, a single accession may adequately represent a named cultivar. However, a single accession cannot capture intraspecific variation in wild species. Accordingly, the PCA and ADMIXTURE results presented here should be interpreted primarily as comparisons among sampled accessions rather than as formal population-level estimates. Broader sampling of wild populations and additional cultivars will be necessary to resolve the ancestry of *R*. ‘Hetian’ more precisely.

### 4.3. Morphological Similarities and a Possible Historical Scenario for Rosa ‘Hetian’

Based on our observations, the morphological characteristics of *R.* ‘Hetian’ resemble those of European old roses rather than hybrid Rugosa roses. *Rosa* ‘Hetian’ has relatively smooth leaflets, fringed sepals, ovate hypanthium and erect hips. By contrast, *R. rugosa* and its cultivars have rugose leaflets, entire sepals, depressed-globose hypanthium and drooping hips. Among European old garden roses, the morphology of *R.* ‘Hetian’ is most consistent with that of Gallica roses. Shared traits include short, slender, dense growth; sparse prickles on the upper part of branchlets; deep purple flowers borne in small clusters; and short styles. *Rosa gallica* is native to central and southern Europe to the Caucasus region, and it has been regarded as one of the earliest roses cultivated in Europe. A selected variety, *R. gallica* ‘Officialis’, was widely cultivated in Europe for culinary and medical use in ancient times [33]. Therefore, we hypothesize that cultivated Gallica roses reached Hetian along the ancient trade routes, such as the Silk Road, and hybridization may have occurred during its dispersal. More comprehensive sampling for local oil-bearing rose cultivars and wild roses along the region related to the Silk Road, as well as archeological information, would produce more evidence for this hypothesis. A comparable pattern has been reported for China roses. As reblooming China roses originated in Ya’an, Sichuan, China, genetic contributions from *R. multiflora* var. *cathayensis*, *R. kwangtungensis* and *R. gigantea* were incorporated into many cultivars, contributing to the diversification of China Roses and Tea Roses [5].

## 5. Conclusions

Our nuclear genomic analyses indicate that *Rosa* ‘Hetian’ is genetically more closely associated with European Old Garden Roses than with Chinese Hybrid Rugosa roses. Among the sampled accessions, *R*. ‘Hetian’ showed the strongest genomic affinity to Gallica roses, with weaker affinities to other Eurasian rose species or cultivars. Our results suggest that a Gallica-related lineage may have substantially shaped the genetic background of R. ‘Hetian’, with additional affinity to other Eurasian rose species and cultivars. We hypothesize that its introduction into Xinjiang occurred through historical dispersal along trans-Eurasian trade routes, possibly including the Silk Road; however, this scenario remains plausible but untested. These findings provide a genomic framework for further investigation of the origin, conservation, and utilization of this distinctive local rose germplasm.

## Figures and Tables

**Table 1:** Plant materials from Genus *Rosa* included in the research.

Type *	Species or Cultivars	Abbr.	Geographic Origin
-	*Rosa* ‘Hetian’	hetian	Hotan Prefecture, Xinjiang, China
Cin.	*R. laxa*	laxa	Xinjiang [C. Asia, C. Siberia]
Cin.	*R. albertii*	albertii	Gansu, Qinghai, Xinjiang [Kazakhstan, W. Siberia]
Cin.	*R. fedtschenkoana*	fedtsche	Xinjiang [Kazakhstan]
Syn.	*R. phoenicia*	phoenicia	[E. Medit. to N. Iraq.]
Gal.	*R. gallica*	gallica	[E. Central Europe and E. Europe and Caucasus]
Gal.	*R. gallica* ‘officinalis’	gal_off	Cultivar from Europe
Gal.	*R.* ‘Belle Isis’	gal_beis	Cultivar bred in Belgium (1845)
Dam.	*R.* ‘Ispahan’	Ispahan	a hybrid of Middle Eastern origin
Dam.	*R.* × *damascena* ‘Summer Damask’	sum_dam	a hybrid of Middle Eastern origin
Dam.	*R.* × *damascena* ‘Autumn Damask’	aut_dam	a hybrid of Middle Eastern origin
Alba	*R*. × *alba*	alba	a hybrid of Asia Minor/eastern Mediterranean origin
Cen.	*R*. × *centifolia*	cent_1	a hybrid of Europe–western Asia region
Cen.	*R*. × *centifolia*	cent_2	a hybrid of Europe–western Asia region
Dam.	*R.* × *damascena* ‘grasse’	grasse	a hybrid of Middle Eastern origin
Syn.	*R. moschata*	moschata	[S. Iran to Afghanistan]
Can.	*R. canina*	canina	[Madeira, N.W. Africa, Europe to Central Asia and Pakistan]
Dam.	*R.* × *damascena* ‘Kazanlashka’	dmt	a hybrid of Middle Eastern origin
Dam.	*R.* × *damascena* ‘koonepamapka’	dmk	a hybrid of Middle Eastern origin
Dam.	*R.* × *damascena* ‘Luogang’	dml	a hybrid of Middle Eastern origin
Rug.	*R. rugosa*	rugosa	E. Jilin, Liaoning, N.E. Shandong [Japan, Korea, Far East]
Rug.	*R. rugosa* ‘Plena’	plena	Cultivar from Northern China
Rug.	*R.* ‘Kushui’	kushui	A natural hybrid from Gansu (Kushui), China
Alba	*R*. × *alba* ‘Baobai’	baobai	a hybrid of Asia Minor/eastern Mediterranean origin
Rug.	*R.* ‘Zizhi’	zizhi	Cultivar bred in Pingyin, Shandong, China (1980)
Rug.	*R.* ‘Thérèse Bugnet’	t_bugnet	Cultivar bred in Canada (1950)
Cin.	*R. davurica*	davurica	North and Northeast China [Japan, Korea, S. Mongolia, E. Siberia]
Rug.	*R. rugosa* ‘Fenghua’	fenghua	Cultivar bred in Pingyin, Shandong, China (1980)
Gal.	*R.* ‘Charles de Mills’	char_dm	Cultivar bred in Netherlands (before 1786)
Gal.	*R.* ‘Duchesse De Montebello’	dd_mont	Cultivar bred in France (1824)
Syn.	*R. arvensis*	arvensis	Europe to Türkiye
Cin.	*R. beggeriana*	beggeriana	Gansu, Xinjiang [Iran to Mongolia and W. Himalaya]
Syn.	*R. brunonii*	brunonii	N.E. Afghanistan to China and N. Myanmar
Dam.	*R.* × *damascena* III	dm3	a hybrid of Middle Eastern origin
Cin.	*R. davurica* var. *glabra*	davu_gla	Heilongjiang, Jilin, Liaoning [N. Korea]
Cin.	*R. glauca*	glauca	Central and S. Europe to European Russia
Out.	*R. hugonis*	hugonis	Gansu, Qinghai, Shaanxi, Shanxi, Sichuan
Out.	*Fragaria* × *ananassa*	Fragaria	a natural hybrid from France

Note: 1. Five resequencing datasets and four plastid genomes were retrieved from NCBI database. Resequencing datasets: *R. laxa*, SRX20385657; *R. fedtschenkoana*, SRX10377633; *R. phoenicia*, SRX10377606; *R. canina*, SRX10377688 and *R. glauca*, SRX10377490. Plastid genomes: *R. fedtschenkoana*, PV786060; *R. canina*, PP976078; *R. hugonis*, PP191172 and *Fragaria* × *ananassa*, OP723290. *R. glauca* was not included in the plastid genome tree. 2. The “Type *” column shows either the taxonomic section of a wild species or the horticultural group of a variety. Abbreviations: Cin. = Sect. *Cinnamomeae*, Syn. = Sect. *Synstylae*, Can. = Sect. *Caninae*, Gal. = Gallica, Dam. = Damask, Cen. = Centifolia, Rug. = Hybrid Rugosa (*R. rugosa* biologically belongs to Sect. *Cinnamomeae*). Moreover, Out. represents outgroup. Taxonomy of species roses follows *Flora Reipublicae Popularis Sinicae* and classification of varieties refers to helpmefind.com.

## Data Availability

The accession links of resequencing data and aligned plastid genomes are presented in Table S1.

## Supplementary Materials

The following supporting information can be downloaded at: https://www.mdpi.com/article/10.3390/genes17101234/s1, Figure S1: Plastid genome of *Rosa* ‘Hetian’; Figure S2: ADMIXTURE results for the *Rosa* accessions included in this study; Table S1: Detailed information on the Rosa accessions, including source, ploidy, plastid genome length, and coverage of the reference sequence.

## References

[1] Xu R., Wang Y., Yuan X., Wang H., Ma S., Liu T., Ma C. (2026). Habitat-driven high-value specialty resource: A review of quality formation mechanisms, functional properties, and industrial development of Hetian rose. J. Beijing For. Univ..

[2] Iwata H., Kato T., Ohno S. (2000). Triparental origin of Damask roses. Gene.

[3] Manners M.M., Morvillo N., Frederick C., Wagner A. (2004). RAPD-PCR answers some long-standing questions about rose identification. XXVI International Horticultural Congress: IV International Symposium on Taxonomy of Cultivated Plants.

[4] Alioua A., Heitzler P. (2023). On the Origin of Cultivated Roses: DNA Authentication of the Bourbon Rose Founding Pedigree. Int. J. Plant Biol..

[5] Zhang C., Jiang Z.-Z., Yang S.-Z., Li S.-Q., Liang Z.-L., Gao X.-F. (2026). Molecular investigation of the progenitors, origin and domestication patterns of diploid Chinese old garden roses. Ann. Bot..

[6] Zhao F., Wang Y., Zhang Y.-J., Sun K., Da L.-L., Zhang H. (2024). Molecular and morphological evidences for the hybrid origin of Kushui Rose. Genet. Resour. Crop Evol..

[7] Cui W.-H., Du X.-Y., Zhong M.-C., Fang W., Suo Z.-Q., Wang D., Dong X., Jiang X.-D., Hu J.-Y. (2022). Complex and reticulate origin of edible roses (*Rosa*, Rosaceae) in China. Hortic. Res..

[8] Debray K., Marie-Magdelaine J., Ruttink T., Clotault J., Foucher F., Malécot V. (2019). Identification and assessment of variable single-copy orthologous (SCO) nuclear loci for low-level phylogenomics: A case study in the genus *Rosa* (Rosaceae). BMC Evol. Biol..

[9] Herklotz V., Zhang M., Nascimento T., Kalfusová R., Lunerová J., Fuchs J., Harpke D., Huettel B., Pfordt U., Wissemann V. (2025). Bimodal centromeres in pentaploid dogroses shed light on their unique meiosis. Nature.

[10] Zhang X., Lan L., Yang Y., Guan H., Peng D., Jiang H., Wu Q., Huang R., Liao X., Lin S. (2026). Pangenomic analyses of rose uncover widespread structure variation and empower genomics-directed breeding. Nat. Genet..

[11] Zhang Z., Yang T., Liu Y., Wu S., Sun H., Wu J., Li Y., Zheng Y., Ren H., Yang Y. (2024). Haplotype-resolved genome assembly and resequencing provide insights into the origin and breeding of modern rose. Nat. Plants.

[12] Song Y., Wang H. (2022). Genetic background analysis of ‘Hetian’ rose based on DNA sequence. Chin. Agric. Sci. Bull..

[13] Wang H. (2013). Germplasm Resources Classification and Evaluation of Oil-Bearing Rose in China Based on Genetic Diversity. Ph.D. Thesis.

[14] Ning H., Lu J., Lu C., Wen H., Nurpolati A., Haibier H., Liu G. (2022). ITS2-based phylogenetic analysis of *Rosa damascena* ‘Hetian’. Mod. Chin. Med..

[15] Allen G.C., Flores-Vergara M.A., Krasynanski S., Kumar S., Thompson W.F. (2006). A modified protocol for rapid DNA isolation from plant tissues using cetyltrimethylammonium bromide. Nat. Protoc..

[16] Chen S., Zhou Y., Chen Y., Gu J. (2018). fastp: An ultra-fast all-in-one FASTQ preprocessor. Bioinformatics.

[17] Prjibelski A., Antipov D., Meleshko D., Lapidus A., Korobeynikov A. (2020). Using SPAdes De Novo Assembler. Curr. Protoc. Bioinform..

[18] Wick R.R., Schultz M.B., Zobel J., Holt K.E. (2015). Bandage: Interactive visualization of de novo genome assemblies. Bioinformatics.

[19] Li H. (2009). Fast and Accurate Short Read Alignment with Burrows-Wheeler Transform. Bioinformatics.

[20] Li H., Handsaker B., Wysoker A., Fennell T., Ruan J., Homer N., Marth G., Abecasis G., Durbin R., Subgroup G.P.D.P. (2009). The Sequence Alignment/Map format and SAMtools. Bioinformatics.

[21] Qu X.-J., Moore M.J., Li D.-Z., Yi T.-S. (2019). PGA: A software package for rapid, accurate, and flexible batch annotation of plastomes. Plant Methods.

[22] Katoh K., Standley D.M. (2013). MAFFT Multiple Sequence Alignment Software Version 7: Improvements in Performance and Usability. Mol. Biol. Evol..

[23] Castresana J. (2000). Selection of Conserved Blocks from Multiple Alignments for Their Use in Phylogenetic Analysis. Mol. Biol. Evol..

[24] Wong T.K.F., Ly-Trong N., Ren H., Demotte P., Baños H., Roger A.J., Susko E., Bielow C., De Maio N., Goldman N. (2026). IQ-TREE 3: Phylogenomic inference software using complex evolutionary models. Mol. Biol. Evol..

[25] McKenna A., Hanna M., Banks E., Sivachenko A., Cibulskis K., Kernytsky A., Garimella K., Altshuler D., Gabriel S., Daly M. (2010). The Genome Analysis Toolkit: A MapReduce framework for analyzing next-generation DNA sequencing data. Genome Res..

[26] Chang C.C., Chow C.C., Tellier L.C.A.M., Vattikuti S., Purcell S.M., Lee J.J. (2015). Second-generation PLINK: Rising to the challenge of larger and richer datasets. GigaScience.

[27] Danecek P., Auton A., Abecasis G., Albers C.A., Banks E., DePristo M.A., Handsaker R.E., Lunter G., Marth G.T., Sherry S.T. (2011). The variant call format and VCFtools. Bioinformatics.

[28] Cheng B., Zhao K., Zhou M., Bourke P.M., Zhou L., Wu S., Sun Y., Geng L., Du W., Yang C. (2025). Phenotypic and genomic signatures across wild *Rosa* species open new horizons for modern rose breeding. Nat. Plants.

[29] Price M.N., Dehal P.S., Arkin A.P. (2010). FastTree 2—Approximately maximum-likelihood trees for large alignments. PLoS ONE.

[30] Yang J., Lee S.H., Goddard M.E., Visscher P.M. (2011). GCTA: A tool for genome-wide complex trait analysis. AM J. Hum. Genet..

[31] Alexander D.H., Novembre J., Lange K. (2009). Fast model-based estimation of ancestry in unrelated individuals. Genome Res..

[32] Bai M., Liu J., Fan C., Chen Y., Chen H., Lu J., Sun J., Ning G., Wang C. (2021). KSN heterozygosity is associated with continuous flowering of *Rosa rugosa* Purple branch. Hortic. Res..

[33] Clovis P. (2023). Rosa gallica L. and Other Gallic Roses: Origin(s) and Role in the Genesis of Cultivated Roses. Ph.D. Thesis.

