# Supplementary material for: Genetic Background of the Hetian Rose Inferred from SNPs in Single-Copy Nuclear Loci and Plastid Genomes
Source: Genes (Basel). 2026 Oct 6;17(10):1234. doi: 10.3390/genes17101234 (PMC13704192; doi:10.3390/genes17101234)
Supplement: Supplementary file 1 [file genes-17-01234-s001.zip › Figure_S1 The plastid genome of Rosa 'Hetian'.pdf]

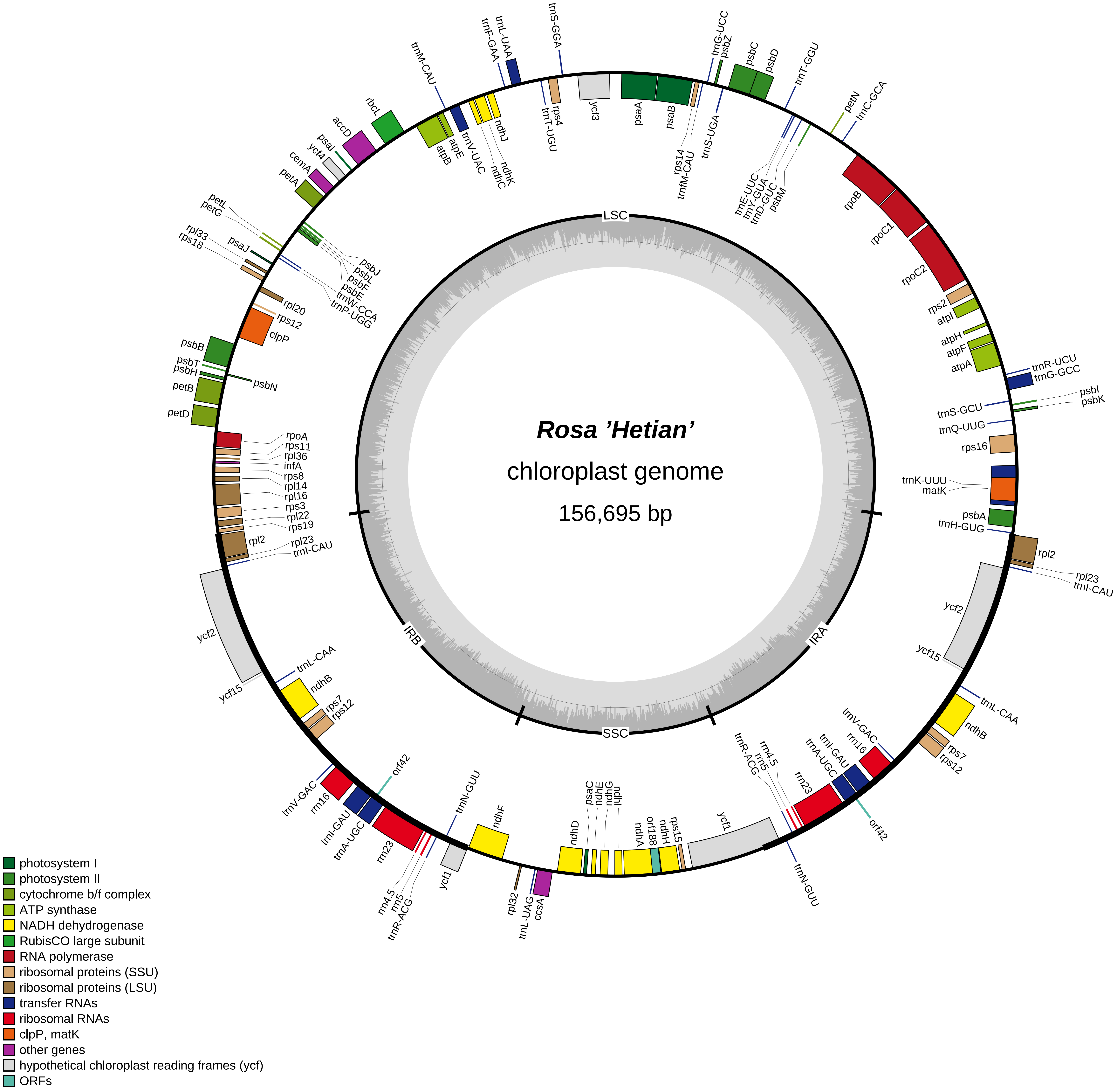

Rosa ’Hetian’
chloroplast genome
156,695 bp
LSC
IRA
IRB
SSC
trnS-GGA
trnL-UAA
trnF-GAA
trnM-CAU
rbcL
accD
psaI
ycf4
cemA
petA
petL
petG
rpl33
rps18
psaJ
psbJ
psbL
psbF
psbE
trnW-CCA
trnP-UGG
rpl20
rps12
clpP
psbB
psbT
psbH
petB
psbN
petD
rpoA
rps11
rpl36
infA
rps8
rpl14
rpl16
rps3
rpl22
rps19
rpl2
rpl23
trnI-CAU
ycf2
ycf15
trnL-CAA
ndhB
rps7
rps12
trnV-GAC
rrn16
trnI-GAU
trnA-UGC
rrn23
orf42
rrn4.5
rrn5
trnR-ACG
trnN-GUU
ycf1
ndhF
rpl32
trnL-UAG
ccsA
ndhD
psaC
ndhE
ndhG
ndhI
ndhA
orf188
ndhH
rps15
ycf1
trnN-GUU
trnR-ACG
rrn4.5
rrn5
rrn23
trnA-UGC
trnI-GAU
rrn16
trnV-GAC
orf42
rps7
rps12
ndhB
trnL-CAA
ycf15
ycf2
rpl23
trnI-CAU
rpl2
trnH-GUG
psbA
trnK-UUU
matK
rps16
trnQ-UUG
trnS-GCU
psbI
psbK
trnR-UCU
trnG-GCC
atpA
atpF
atpH
atpI
rps2
rpoC2
rpoC1
rpoB
trnC-GCA
petN
trnT-GGU
psbM
trnD-GUC
trnY-GUA
trnE-UUC
psbD
psbC
psbZ
trnG-UCC
trnS-UGA
trnfM-CAU
rps14
psaB
psaA
ycf3
rps4
trnT-UGU
ndhJ
ndhK
ndhC
trnV-UAC
atpE
atpB
photosystem I
photosystem II
cytochrome b/f complex
ATP synthase
NADH dehydrogenase
RubisCO large subunit
RNA polymerase
ribosomal proteins (SSU)
ribosomal proteins (LSU)
transfer RNAs
ribosomal RNAs
clpP, matK
other genes
hypothetical chloroplast reading frames (ycf)
ORFs
