# Supplementary material for: Genetic Background of the Hetian Rose Inferred from SNPs in Single-Copy Nuclear Loci and Plastid Genomes
Source: Genes (Basel). 2026 Oct 6;17(10):1234. doi: 10.3390/genes17101234 (PMC13704192; doi:10.3390/genes17101234)
Supplement: Supplementary file 1 [file genes-17-01234-s001.zip › Figure_S2 Admixture results of roses involved in this study, including Rosa 'Hetian'.pdf]

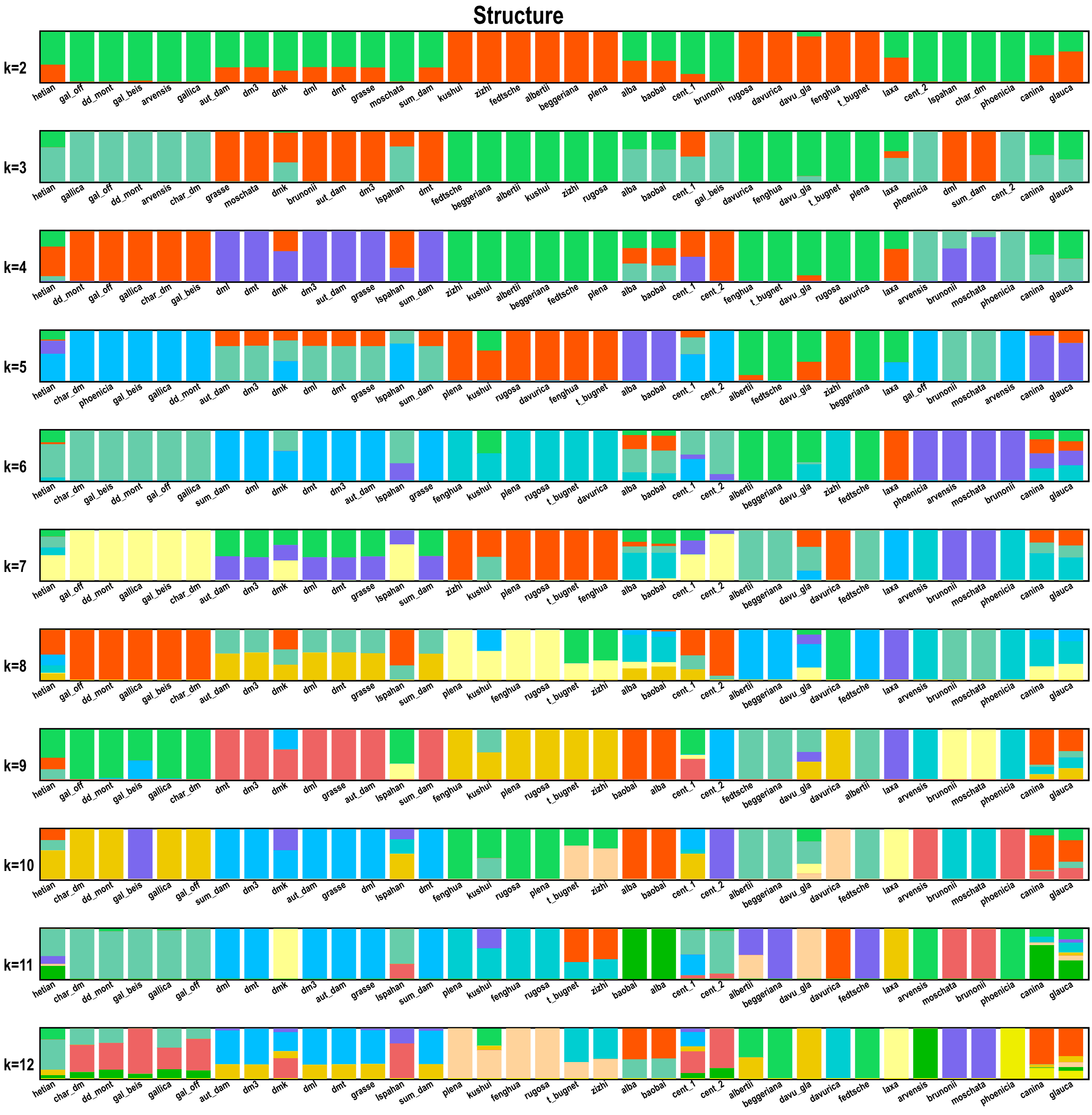
Structure
k=2
hetian gal_off dd_mont gal_beis arvensis gallica aut_dam dm3 dmk dml dmt grasse moschata sum_dam kushui zizhi fedtsche albertii beggeriana plena alba baobai cent_1 brunonii rugosa davurica davu_gla fenghua t_bugnet laxa cent_2 lspahan char_dm phoenicia canina glauca
k=3
hetian gallica gal_off dd_mont arvensis char_dm grasse moschata dmk brunonii aut_dam dm3 lspahan dmt fedtsche beggeriana albertii kushui zizhi rugosa alba baobai cent_1 gal_beis davurica fenghua davu_gla t_bugnet plena laxa phoenicia dml sum_dam cent_2 canina glauca
k=4
hetian dd_mont gal_off gallica char_dm gal_beis dml dmt dmk dm3 aut_dam grasse lspahan sum_dam zizhi kushui albertii beggeriana fedtsche plena alba baobai cent_1 cent_2 fenghua t_bugnet davu_gla rugosa davurica laxa arvensis brunonii moschata phoenicia canina glauca
k=5
hetian char_dm phoenicia gal_beis gallica dd_mont aut_dam dm3 dmk dml dmt grasse lspahan sum_dam plena kushui rugosa davurica fenghua t_bugnet alba baobai cent_1 cent_2 albertii fedtsche davu_gla zizhi beggeriana laxa gal_off brunonii moschata arvensis canina glauca
k=6
hetian char_dm gal_beis dd_mont gal_off gallica sum_dam dml dmk dmt dm3 aut_dam lspahan grasse fenghua kushui plena rugosa t_bugnet davurica alba baobai cent_1 cent_2 albertii beggeriana davu_gla zizhi fedtsche laxa phoenicia arvensis moschata brunonii canina glauca
k=7
hetian gal_off dd_mont gallica gal_beis char_dm aut_dam dm3 dmk dml dmt grasse lspahan sum_dam zizhi kushui plena rugosa t_bugnet fenghua alba baobai cent_1 cent_2 albertii beggeriana davu_gla davurica fedtsche laxa arvensis brunonii moschata phoenicia canina glauca
k=8
hetian gal_off dd_mont gallica gal_beis char_dm aut_dam dm3 dmk dml dmt grasse lspahan sum_dam plena kushui fenghua rugosa t_bugnet zizhi alba baobai cent_1 cent_2 albertii beggeriana davu_gla davurica fedtsche laxa arvensis brunonii moschata phoenicia canina glauca
k=9
hetian gal_off dd_mont gal_beis gallica char_dm dmt dm3 dmk dml grasse aut_dam lspahan sum_dam fenghua kushui plena rugosa t_bugnet zizhi baobai alba cent_1 cent_2 fedtsche beggeriana davu_gla davurica albertii laxa arvensis brunonii moschata phoenicia canina glauca
k=10
hetian char_dm dd_mont gal_beis gallica gal_off sum_dam dm3 dmk aut_dam grasse dml lspahan dmt fenghua kushui rugosa plena t_bugnet zizhi alba baobai cent_1 cent_2 albertii beggeriana davu_gla davurica fedtsche laxa arvensis brunonii moschata phoenicia canina glauca
k=11
hetian char_dm dd_mont gal_beis gallica gal_off dml dmt dmk dm3 aut_dam grasse lspahan sum_dam plena kushui fenghua rugosa t_bugnet zizhi baobai alba cent_1 cent_2 albertii beggeriana davu_gla davurica fedtsche laxa arvensis moschata brunonii phoenicia canina glauca
k=12
hetian char_dm dd_mont gal_beis gallica gal_off aut_dam dm3 dmk dml dmt grasse lspahan sum_dam plena kushui fenghua rugosa t_bugnet zizhi alba baobai cent_1 cent_2 albertii beggeriana davu_gla davurica fedtsche laxa arvensis brunonii moschata phoenicia canina glauca
